# Workplace Violence and Job Performance among Community Healthcare Workers in China: The Mediator Role of Quality of Life

**DOI:** 10.3390/ijerph121114872

**Published:** 2015-11-20

**Authors:** Wei-Quan Lin, Jiang Wu, Le-Xin Yuan, Sheng-Chao Zhang, Meng-Juan Jing, Hui-Shan Zhang, Jia-Li Luo, Yi-Xiong Lei, Pei-Xi Wang

**Affiliations:** 1 Department of Preventive Medicine, School of Public Health, Guangzhou Medical University, Guangzhou 510182, China; E-Mails: linweiquan0503@163.com (W.-Q.L.); zhanghuishan_25@163.com (H.-S.Z.); baby.luo.2008@163.com (J.-L.L.); 2 Baoan Center Hospital of Shenzhen, 6 Xiyuan Road, Baoan District, Shenzhen 518102, China; E-Mails: WuJiang731@163.com (J.W.); pocoo@126.com (S.-C.Z.); 3 Department of Nursing, School of Nursing, Guangzhou Medical University, Guangzhou 510182, China; E-Mail: yuanlexin2015@163.com; 4 Institute of Public Health, School of Nursing, Henan University, Kaifeng 475004, China; E-Mail: jing53905@163.com

**Keywords:** community healthcare worker, workplace violence, job performance, quality of life, mediator

## Abstract

*Objective*: To explore the impact of workplace violence on job performance and quality of life of community healthcare workers in China, especially the relationship of these three variables. *Methods*: From December 2013 to April 2014, a total of 1404 healthcare workers were recruited by using the random cluster sampling method from Community Health Centers in Guangzhou and Shenzhen. The workplace violence scale, the job performance scale and the quality of life scale (SF-36) were self-administered. The structural equation model constructed by Amos 17.0 was employed to assess the relationship among these variables. *Results*: Our study found that 51.64% of the respondents had an experience of workplace violence. It was found that both job performance and quality of life had a negative correlation with workplace violence. A positive association was identified between job performance and quality of life. The path analysis showed the total effect (*β* = −0.243) of workplace violence on job performance consisted of a direct effect (*β* = −0.113) and an indirect effect (*β* = −0.130), which was mediated by quality of life. *Conclusions*: Workplace violence among community healthcare workers is prevalent in China. The workplace violence had negative effects on the job performance and quality of life of CHCs’ workers. The study suggests that improvement in the quality of life may lead to an effective reduction of the damages in job performance caused by workplace violence.

## 1. Introduction

The New Health Care Reform Plan issued by the Chinese government in 2009 re-emphasized the central role of Community Health Centers (CHCs) in providing cost-effective and convenient primary care to the public, which aimed to improve equitable access to basic healthcare for its residents by building a strong, primary care-based delivery system [1]. To achieve the above goals, there is an urgent need to promote the work status and health status of community healthcare workers [2]. As the population ages and lifestyle changes, CHCs play a much more significant role in the healthcare system, and CHC healthcare workers, as the main pillar of primary care providers, should take on more workload than before.

The current tense physician-patient relationship particularly caused by workplace violence is widely recognized to be an exigent social problem that might impact the health status and the work status of healthcare workers in CHCs. The issue of workplace violence used to be a hot research topic of public health [3,4,5]. Workplace violence is the intentional use of physical force or power, such as physical assaults and threats of assaults, directly towards people at work or on duty [6]. Workplace violence, as an occupational hazard in the healthcare setting, can lead to a variety of adverse consequences for the victims, including anger, anxiety, depression, fear, sleep disruption, job strain, job dissatisfaction and job turnover of health workers [7,8,9,10]. It is demonstrated that the incidence of workplace violence against medical workers in general hospitals is only about 9.5% in the U.K., but this study only reports physical violence [11]. The situation is more serious in the USA and Turkey: 78% [12] and 87% [13], respectively. Similarly, the incidence rate is about 71% in China [14]. Besides, some studies had proven that workplace violence influences employee’s work status, like job performance [15,16]. Schermerhorm has defined job performance as the quality and quantity of tasks of an individual or a group, which also has been called staff productivity [17]. In a previous study, Schat’s research confirmed that U.S. workers’ job performance was damaged by workplace violence [15]. However, few studies have been conducted to investigate the situation in primary care facilities. Additionally, studies examining the association between workplace violence and job performance in primary care settings are rare.

In addition, quality of life (QOL) has been introduced to estimate people’s health status, which is defined as an individual’s satisfaction or happiness with the eight dimensions of life [18]. Teles’s study demonstrated that QOL was decreased by workplace violence [19]. Not only does QOL relate to an individual’s own mental and physical health, but it significantly influences the quality and safety of the health services that they provide [20]. Studies have identified the relationship between workplace violence and QOL [20,21], as well as the association between QOL and job performance [22]. However, few studies have performed research on the triadic connections in CHC healthcare workers in China. There might especially be a spatial relationship between those.

Therefore, this study tries to investigate the current status of workplace violence in primary care settings in China and probes into the relationship of these three variables. Last, we attempt to analyze the mechanism of how workplace violence affects job performance. One hypothesis is that there might a spatial relationship between workplace violence and job performance, which might be mediated by QOL.

## 2. Methods

### 2.1. Respondents and Procedure

This was a cross-sectional study conducted in Guangzhou and Shenzhen, China. Between December 2013 and April 2014, 26 and 63 CHCs were firstly selected as the study settings in Guangzhou and Shenzhen by using a simple random sampling method. Then, employing a cluster sampling method, 1626 health workers were recruited. A research assistant sent the questionnaires to the respondents. The questionnaires were self-administered. The research assistant briefly informed the respondents about the purpose and significance of the study. Information sheets on the participants’ rights were given along with written informed consent. The completed questionnaires were double checked to see if there was any missing data. In total, 1404 respondents (711 from Guangzhou and 693 from Shenzhen) completed the questionnaires with a response rate of 86.43%. This study was approved by the ethics committee of Guangzhou Medical University.

### 2.2. Instruments

#### 2.2.1. Workplace Violence Scale

The workplace violence scale (WVS) developed by Wang was adapted and used to evaluate the healthcare workers’ frequency of suffering from workplace violence [7,23]. The scale was divided into five dimensions (one item for each dimension, 5 items in total), including physical assault (PA), emotional abuse (EA), threat (T), verbal sexual harassment (VSH) and sexual assault (SA). For consistency in responses, all items were represented by four points to reflect the frequencies of violence. One illustration of the items was “In the past 12 months, have you suffered from a physical assault in the workplace, which includes being spit on, bitten, hit, or pushed? (0 = never, 1 = 1 time, 2 = 2~3 times, 3 = ≥4 times)”. The scale score was created by adding the scores for each item, ranging from 0 to 15, with higher scores indicating a higher frequency of experiencing violence. The score for never experienced workplace violence will get a score of 0. In this study, the Cronbach alpha coefficient for the WVS was 0.704.

#### 2.2.2. Job Performance Scale

The job performance scale (JPS) was deployed for the measurement of the job performance of healthcare worker, which was developed by Motowidlo and Scotter [24,25]. It included three dimensions, namely job dedication (JD), task performance (TP) and interpersonal facilitation (IF), which were measured by 16 self-reported items. The items are rated from 1 (strongly disagree) to 6 (strongly agree). The scale scores of JPS were the sum of these 16 items (range: 16 to 96); for instance, “I am voluntarily taking a challenging job” and “I have good co-operation with other colleagues.” Our study showed that the Cronbach alpha coefficient of the JPS was 0.942, and those of the three sub-scales were 0.834, 0.919 and 0.934.

#### 2.2.3. Quality of Life Scale

The quality of life (QOL) scale reflects health status. QOL was measured by SF-36, which was developed by Boston: New England Medical Center, The Health Institute [18]. SF-36 consisted of 36 items, which were classified into 8 dimensions, *i.e.*, physical functioning (PF), role limitations due to physical problems (RP), bodily pain (BP), general health (GH), vitality (VT), social functioning (SF), role limitations due to emotional problems (RE) and mental health (MH). Take some items, for example: “In general, would you say your health is: 1 = excellent, 2 = very good, 3 = good, 4 = fair, 5 = poor?” and “I expect my health to get worse: 1 = definitely true, 2 = mostly true, 3 = don’t know, 4 = mostly false, 5 = definitely false.” Scores for each dimension were coded and added up and then were translated into a scale score ranging from 0 to 100 [18]. Generally, a higher score prompted a better health status [26]. Studies have shown that SF-36 has good reliability and validity for health status measurement in China [26,27]. In this study, the Cronbach alpha coefficient for the QOL scale was 0.792.

### 2.3. Data Analysis

In the present study, the Statistical Package for Social Sciences (SPSS), Version 17.0 (SPSS, Inc., Chicago, IL, USA), was used for statistical analysis. Data were presented as the mean ± the standard deviation (sd) of continuous variables and n (%) for categorical variables. The chi square test was used to compare the incidence of workplace violence among the respondents with different socio-demographic characteristics. The *t*-test was employed to compare the dimension scores of each scale between the respondents with and without experiences of workplace violence. Correlation analysis, regression analysis and path analysis were used to examine the relationship among the three variables, including workplace violence, job performance and QOL. A *p*-value <0.05 was considered statistically significant.

The structural equation model (SEM) for path analysis was constructed by the AMOS 17.0 program to analyze the effect of workplace violence on job performance and QOL. A model was established with workplace violence as the independent variable, job performance as the dependent variable and QOL as the mediating variable. The model was considered to have a good fit when all path coefficients were significant at the level of 0.05; χ^2^/df, was below 5; the standardized root mean square residual (SRMR) was below 0.08; the root mean square error of approximation (RMSEA) was below 0.08; as well as the goodness-of-fit index (GFI), the normed fit index (NFI), the Tacker–Lewis index (TFI) and the comparative fit index (CFI) were ≥0.95 [28].

## 3. Results

Of the 1404 respondents, about three quarters were female (73.29%). Approximately one half was aged 30~40 years old. About 76.21% of the respondents described themselves as married (Table 1). More than half of the respondents had an education level of college or above. About 40% of the respondents were GPs, whilst another 40% were nurses. Most of them were fixed-term workers in CHCs. Almost 70% of the respondents had a monthly income between RMB 2000 and 6000, which was equivalent to the area’s median income.

In the past 12 months, more than half of the respondents (51.64%) experienced workplace violence (Table 1). The incidence of PA was 9.69%; EA was 46.23%; T was 23.08%; VSH was 10.54%; and SA was 4.34%. The chi square test showed that the incidence of workplace violence had no significant difference among the respondents with different socio-demographic characteristics, including gender and marital status. However, significant differences did exist in age (*p* = 0.005), education level (*p* = 0.001), occupation (*p* = 0.003) and monthly income (*p* = 0.002).

There were significant differences in the job performance between the respondents who experienced workplace violence and those who did not (*p* < 0.001) (Table 2). Significant differences were also identified in the three dimensions used to measure job performance, including JD, TP and IF. As for QOL, the respondents who did not experience workplace violence had a higher QOL score when compared to those who had experienced it. Similar findings were observed for the eight dimensions under QOL, as well.

A correlation matrix for the study variables is presented in Table 3. It was shown that workplace violence was negatively related to job performance (*r* = −0.205, *p* < 0.001), whilst there was a significantly negative correlation between workplace violence and QOL (*r* = −0.313, *p* < 0.001). However, a positive correlation was identified between job performance and QOL (*r* = 0.365, *p* < 0.001).

Regression analysis among variables is presented in Table 4. The effect of workplace violence on job performance, including its three dimensions, was examined. Results showed that workplace violence had a relatively negative predictive effect on job performance (*β* = −0.205, *p* < 0.001), job dedication (*β* = −0.197, *p* < 0.001), task performance (*β* = −0.166, *p* < 0.001) and interpersonal facilitation (*β* = −0.181, *p* < 0.001) of healthcare workers in CHCs. The effect of workplace violence on the quality of life was explored, and a relatively negative predictive effect was reported (*β* = −0.313, *p* < 0.001). The effect of workplace violence and quality of life on job performance was also tested, and the standardized regression coefficients were *β* = −0.100 and *β* = 0.333, respectively (all *p* < 0.001).

**Figure 1:**
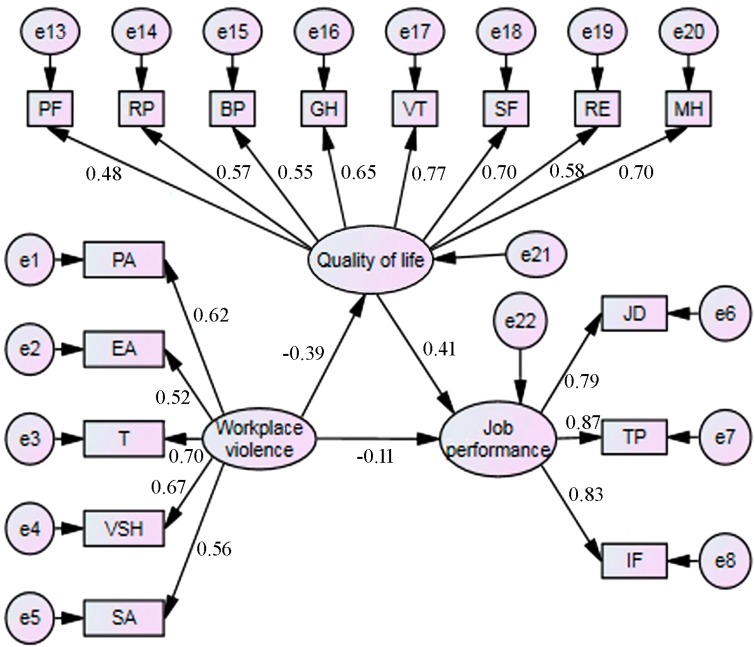
The original model. (*β*: standardized path coefficient. The direct effect: *β* = −0.105, workplace violence → job performance. The indirect effect: *β* = −0.160, workplace violence → quality of life → job performance. The total effect: *β* = −0.26, workplace violence on job performance, consisted of a direct effect (*β* = −0.105) and an indirect effect (*β* = −0.160), which was mediated by quality of life. PA: physical assault; EA: emotional abuse; T threat; VSH: verbal sexual harassment; SA: sexual assault. JD: job dedication; TP: task performance; IF: interpersonal facilitation. PF: physical functioning; RP: role limitations due to physical problems; BP: bodily pain; GH: general health; VT: vitality; SF: social functioning; RE: role limitations due to emotional problems; MH: mental health.)

**Figure 2:**
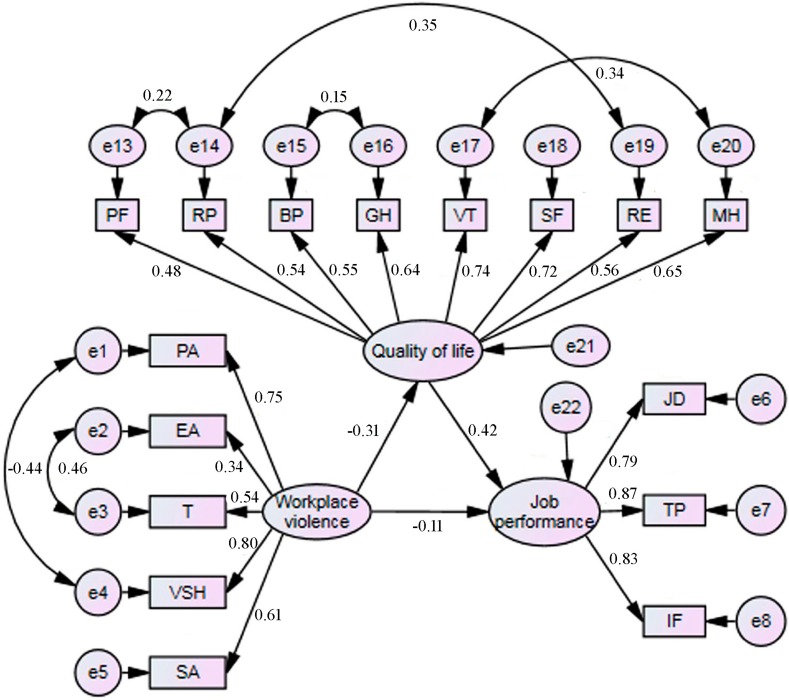
The final model. (*β*: standardized path coefficient. The direct effect: *β* = −0.113, workplace violence → job performance. The indirect effect: *β* = −0.130, workplace violence → quality of life → job performance. The total effect: *β* = −0.243, workplace violence on job performance, consisted of a direct effect (*β* = −0.113) and an indirect effect (*β* = −0.130), which was mediated by quality of life. PA: physical assault; EA: emotional abuse; T threat; VSH: verbal sexual harassment; SA: sexual assault. JD: job dedication; TP: task performance; IF: interpersonal facilitation. PF: physical functioning; RP: role limitations due to physical problems; BP: bodily pain; GH: general health; VT: vitality; SF: social functioning; RE: role limitations due to emotional problems; MH: mental health.)

Path analysis on the original model was performed, which is shown in Figure 1. According to the modification index values, the correlation between EA and T (*r* = 0.548, *p* < 0.001), PA and VSH (*r* = 0.419, *p* < 0.001), PF and RP (*r* = 0.429, *p* < 0.001), RP and RE (*r* = 0.546, *p* < 0.001), BP and GH (*r* = 0.450, *p* < 0.001) and VT and MH (*r* = 0.657, *p* < 0.001), the modified model (final model) was constructed and is shown in Figure 2. Table 5 provides path coefficients between various structural variables. Fit indices of the final model are presented in Table 6, which revealed a good fit of the data.

As can be seen from Figure 2 and Table 5, workplace violence had a negative effect on job performance, which was mediated by QOL. The total effect (*β* = −0.243) of workplace violence on job performance was comprised of not only its direct effect (*β* = −0.113), but also the indirect effect (*β* = −0.130) generated by QOL.

**Table 1:** Basic demographic characteristics of the whole sample and subgroups according to exposure to workplace violence.

	Entire Sample (n = 1404)		Workplace Violence Cases (n = 725) ^a^		Statistics	
	*n*	*%*	*n*	*%*	*χ^2^*	*p*
Gender			725	51.64	1.017	0.334
Male	375	26.71	202	53.87		
Female	1029	73.29	523	50.83		
Age group, years					12.713	0.005
20~29	410	29.20	222	54.15		
30~39	671	47.79	352	52.46		
40~49	241	17.17	124	51.45		
≥50	82	5.84	27	32.93		
Marital status					1.004	0.605
Married	1070	76.21	553	51.68		
Single	310	22.08	162	52.26		
Divorce/widowed	24	1.71	10	41.67		
Education level					14.185	0.001
Professional school	156	11.11	64	41.03		
Junior college	444	31.26	214	48.20		
College or above	804	57.26	447	55.60		
Occupation					11.389	0.003
General practitioner	568	40.46	306	53.87		
Nurse	565	40.24	304	53.81		
Others	271	19.30	115	42.44		
Employment					1.721	0.423
Permanent	526	37.46	275	52.28		
contract	817	58.19	414	50.67		
Other	61	4.34	36	59.02		
Monthly income, RMB					15.349	0.002
<2000	122	8.69	49	40.16		
2000~3999	539	38.39	262	48.61		
4000~5999	444	31.62	238	53.60		
≥6000	299	21.30	176	58.86		

Note: ^a^ A case of workplace violence was defined as the healthcare worker getting a score of at least 1 on the workplace violence scale.

**Table 2:** Univariate analysis between whether or not one experienced workplace violence.

	Entire sample (n = 1404)		Workplace Violence Cases (n = 725) ^a^		Non-Workplace Violence cases (n = 679)		Statistics	
	*m*	*sd*	*m*	*sd*	*m*	*sd*	*t*	*p*
Job performance	76.02	10.22	74.64	10.84	77.48	9.30	−5.257	<0.001
JD	27.11	4.18	26.57	4.32	27.70	3.94	−5.098	<0.001
TP	24.09	3.64	23.69	3.88	24.52	3.32	−4.267	<0.001
IF	24.81	3.66	24.38	3.94	25.27	3.28	−4.585	<0.001
Quality of life	75.60	14.71	71.85	15.55	79.60	12.60	−10.218	<0.001
PF	89.78	11.97	88.57	12.81	91.07	10.86	−3.935	<0.001
RP	77.12	35.22	70.69	37.69	83.98	30.97	−7.194	<0.001
BP	87.95	13.80	85.73	14.74	90.32	12.29	−6.320	<0.001
GH	64.37	19.98	60.82	20.76	68.17	18.40	−7.008	<0.001
VT	66.88	16.52	63.42	16.86	70.57	15.32	−8.293	<0.001
SF	78.22	17.97	74.84	18.67	81.83	16.45	−7.419	<0.001
RE	74.00	37.00	66.80	38.80	81.69	33.34	−7.686	<0.001
MH	66.46	15.30	63.93	15.91	69.15	14.15	−6.481	<0.001

Notes: ^a^ A case of workplace violence case defined as the healthcare worker getting a score of at least 1 on the workplace violence scale. JD: job dedication; TP: task performance; IF: interpersonal facilitation. PF: physical functioning; RP: role limitations due to physical problems; BP: bodily pain; GH: general health; VT: vitality; SF: social functioning; RE: role limitations due to emotional problems; MH: mental health.

**Table 3:** Correlation matrix for the study variables.

	Workplace Violence	Job Performance	Quality of Life
Workplace violence	1.0		
Job performance	−0.205 ***	1.0	
Quality of life	−0.313 ***	0.365 ***	1.0

Note: *** *p* < 0.001.

**Table 4:** Regression analysis among variables.

	Independent Variable	Dependent Variable	*β* ^a^	*t*	*p*
	Workplace violence	Job performance	−0.205	−7.836	<0.001
		Job dedication	−0.197	−7.524	<0.001
		Task performance	−0.166	−6.318	<0.001
		Interpersonal facilitation	−0.181	−6.908	<0.001
	Workplace violence	Quality of life	−0.313	−12.351	<0.001
Workplace violence, Quality of life		Job performance ^b^	−0.100	−3.853	<0.001
			0.333	12.795	<0.001

Notes: ^a^ Standardized regression coefficient. ^b^ The dependent variable was “job performance”; the independent variables were “workplace violence” and “quality of life”.

**Table 5:** The path coefficients between structural variables.

Path			Before Correction			After Correction		
			*β* ^a^	*t*	*p*	*Β* ^a^	*t*	*p*
Quality of life	←	Workplace violence	−0.391	−9.435	<0.001	−0.313	−7.660	<0.001
Job performance	←	Workplace violence	−0.105	−3.099	0.002	−0.113	−3.626	<0.001
Job performance	←	Quality of life	0.410	10.368	<0.001	0.417	10.473	<0.001

Note: ^a^ Standardized path coefficient.

**Table 6:** Fit indices for the structural models ^a^.

	*χ^2^*	*χ^2^/df*	*SRMR*	*RMSEA*	*GFI*	*NFI*	*TFI*	*CFI*
The original model	1077.454	9.598	0.055	0.078	0.948	0.913	0.894	0.921
The final model	405.336	4.267	0.050	0.048	0.965	0.951	0.952	0.962

Note: ^a^ A model is considered to have a good fit if all path coefficients were significant at the level of 0.05; *χ^2^*/df, was below 5; the standardized root mean square residual (SRMR) was below 0.08; the root mean square error of approximation (RMSEA) was below 0.08; as well as the goodness-of-fit index (GFI), the normed fit index (NFI), the Tacker–Lewis index (TFI) and the comparative fit index (CFI) were ≥0.95.

## 4. Discussion

### 4.1. Main Findings

Our study found that more than half of community healthcare workers experienced workplace violence. It was demonstrated that workplace violence negatively affected the QOL and job performance of healthcare workers in CHCs. However, job performance and QOL were positively associated with each other. We found evidence to suggest that there was a mediator role of QOL on the association between workplace violence and job performance.

### 4.2. Comparisons with Previous Findings

Results showed that more than half of community health workers suffered from workplace violence, which is consistent with the findings of previous studies [20]. On the one hand, annual rates of physical aggression against healthcare workers in most studies range between 7% and 12% [5,29,30,31,32], and 9.69% was found by our study. On the other hand, non-physical assaults (EA, T and VSH) were the most frequently experienced by community health workers, which is consistent with the findings of previous studies in Pakistan (72.5%) [33] and in the U.S. (75.0%) [12]. Wells and Bowers supported that bullying and intimidation were the most common form of workplace violence in the U.K. [32]. However, in aggregate (physical aggression, non-physical assaults), this figure is smaller than that reported by Lin, whose study showed that more than 70% of health workers in general hospitals in Shenzhen, China, experienced workplace violence [14]. Our figure is also smaller than that in general hospitals in the U.S. (78%) [12]. One possible explanation of this observation is the smaller number of outpatient consultations and less medical charges in CHCs than in general hospitals.

Our study found that young community health workers were more likely to suffer from workplace violence when compared to their older counterparts, which is possibly due to the reasons like few service hours, unfamiliarity with the environment and poor level of professional skills. The respondents with a high income had more chances to experience workplace violence than those with a low income, which might be attributed to the heavier workload they assumed. The workload might be positively associated with the probability of experiencing workplace violence. This finding might also be due to their being more sensitive to workplace violence. Besides, working in the employer’s house is a potential risk factor for suffering violence, and Hanson’s study found that 61.3% of female homecare workers in the consumer-driven model experienced at least one type of workplace violence in the past year [34].

It was shown that community health workers who experienced workplace violence reported a lower score in each dimension of job performance than those who did not, which suggests that job performance is damaged by workplace violence. Our finding is consistent with the study conducted in the USA by Schat [15]. Similarly, results showed that scores of each dimension under QOL rated by the community health workers who experienced workplace violence were lower than those who did not. This indicates that each dimension of QOL is impacted by workplace violence. Studies by Zeng among psychiatric nurses [35] and by Couto among drivers and conductors [36] showed that EA was positively associated with emotional injury. Workplace violence in CHCs is a significant stressor for community healthcare workers [37]. The stressor might be represented by the emotions of anger, anxiety, fear and depression, leading to a negative impact on job performance and QOL. Therefore, we urgently need to take actions to prevent workplace violence in CHCs.

Results showed that QOL was significantly positively associated with the job performance of health workers in CHCs. A previous study by Mein also found that poor QOL was strongly associated with reduced work performance [38]. Based on this finding, policy makers, including CHC managers, may take steps to improve the QOL of health workers for the improvement of their job performance.

Our study found that workplace violence had a significantly negative predicative effect on the job performance of community health workers, the association of which was mediated by QOL. Our observation is consistent with previous studies [15,16,20,22]. Workplace violence could weaken the job performance of community health workers through damaging their QOL. This finding suggests that interventions aiming to improve the QOL of community health workers may lead to an improvement of their job performance, such as relieving depression and fear by confiding in friends, moderate exercise and keeping health.

In addition, education and training about coping with workplace violence is an important measure for preventing workplace violence according to the U.S. Occupational Safety and Health Administration (OSHA) updated guidelines [39]. Providing education and training is an obvious possibility to increase safety and security, and it was considered to provide it at a national level [40]. Our study showed that workplace violence among community healthcare workers is prevalent, and there is room for improvement to prevent and relieve workplace violence by education and training.

### 4.3. Strengths and Limitations

To the best of our knowledge, this is the first study to explore the mediating role of QOL on the relationship between workplace violence and job performance among community healthcare workers in China. The study by Shahzad suggested that health workers would not disclose their experiences with respect to workplace violence, such as verbal abuse, to their friends or peers, since they perceived that it was useless [41]. Under such a condition, more attention should be paid to how to avoid violence in the workplace against health workers in CHCs. The findings of our study may help to develop effective approaches for reduced workplace violence and improved job performance of community healthcare workers.

In addition, the limitations of the study should be addressed. Firstly, the cross-sectional nature of the current study did not allow us to deduce any cause inferences. Secondly, information bias might be introduced, since all of the data were collected through self-reported questionnaires. Thirdly, some factors that might influence the job performance of community health workers were not included in the current study.

## 5. Conclusions

In conclusion, our study shows that the incidence of workplace violence is high in CHCs in China. Workplace violence is found to have a negative correlation with job performance and QOL, while a positive correlation exists between job performance and QOL. The negative effect of workplace violence on job performance is mediated by the QOL. Except for interventions to avoid workplace violence, those targeting the improvement of QOL might also be effective at improving the job performance of health workers in CHCs in China.

## Abbreviations

CHC, Community Health Center; WVS, workplace violence scale; PA, physical assault; EA, emotional abuse; T, threat; VSH, verbal sexual harassment; SA, sexual assault; JPS, job performance scale; JD, job dedication; TP, task performance; IF, interpersonal facilitation; QOL, quality of life; PF, physical functioning; RP, role limitations due to physical problems; BP, bodily pain; GH, general health; VT, vitality; SF, social functioning; RE, role limitations due to emotional problems; MH, mental health.
